# Antioxidant Effects of Protocatechuic Acid and Protocatechuic Aldehyde: Old Wine in a New Bottle

**DOI:** 10.1155/2021/6139308

**Published:** 2021-11-08

**Authors:** Shijun Zhang, Zhibo Gai, Ting Gui, Juanli Chen, Qingfa Chen, Yunlun Li

**Affiliations:** ^1^First Clinical Medical College, Shandong University of Traditional Chinese Medicine, Jinan 250355, China; ^2^Key Laboratory of Traditional Chinese Medicine for Classical Theory, Ministry of Education, Shandong University of Traditional Chinese Medicine, Jinan 250355, China; ^3^The Institute for Tissue Engineering and Regenerative Medicine, The Liaocheng University/Liaocheng People's Hospital, Liaocheng, China; ^4^Innovation Research Institute of Traditional Chinese Medicine, Shandong University of Traditional Chinese Medicine, Jinan 250355, China; ^5^The Third Department of Cardiovascular Diseases, Affiliated Hospital of Shandong University of Traditional Chinese Medicine, Jinan 250355, China

## Abstract

Phenolic compounds are naturally present as secondary metabolites in plant-based sources such as fruits, vegetables, and spices. They have received considerable attention for their antioxidant, anti-inflammatory, and anti-carcinogenic properties for protection against many chronic disorders such as neurodegenerative diseases, diabetes, cardiovascular diseases, and cancer. They are categorized into various groups based on their chemical structure and include phenolic acids, flavonoids, curcumins, tannins, and quinolones. Their structural variations contribute to their specific beneficial effects on human health. The antioxidant property of phenolic compounds protects against oxidative stress by up-regulation of endogenous antioxidants, scavenging free radicals, and anti-apoptotic activity. Protocatechuic acid (PCA; 3,4-dihydroxy benzoic acid) and protocatechuic aldehyde (PAL; 3,4-dihydroxybenzaldehyde) are naturally occurring polyphenols found in vegetables, fruits, and herbs. PCA and PAL are the primary metabolites of anthocyanins and proanthocyanidins, which have been shown to possess pharmacological actions including antioxidant activity in vitro and in vivo. This review aims to explore the therapeutic potential of PCA and PAL by comprehensively summarizing their pharmacological properties reported to date, with an emphasis on their mechanisms of action and biological properties.

## 1. Introduction

**Figure 1:**
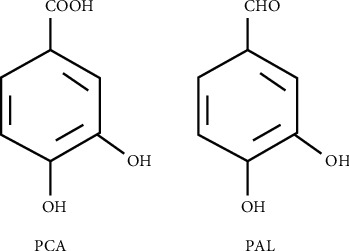
Chemical structure of protocatechuic acid (PCA) and protocatechuic aldehyde (PAL).

Polyphenols are natural compounds found abundantly in plant-based products that have been associated with potential beneficial effects on human health. They play a significant role in many physiological and metabolic processes [1], such as reducing the risk of various diseases including cardiovascular and neurodegenerative diseases, cancer, and diabetes [2, 3] in human beings. Protocatechuic acid (PCA; 3,4-dihydroxybenzoic acid) and protocatechuic aldehyde (PAL; 3,4-dihydroxybenzaldehyde) are the primary metabolites of complex polyphenols [3] present in vegetables, fruits, and herbs. PCA is a water-soluble benzoic acid derivative (Figure 1), reported to have anti-atherosclerotic, anti-inflammatory, antineoplastic, analgesic, antibacterial, hepatoprotective, and antiviral effects in both in vivo and in vitro studies [3-6]. It plays an important role in reversing the biochemical changes induced by cardiac dysfunction and diabetes [7] and reducing the metabolic disorders associated with obesity [8]. PAL—a natural, water-soluble phenolic aldehyde (Figure 1)—is also a naturally occurring compound resulting from phenolic acids' degradation [9]. PAL is reported to have antiadipogenic, anti-proliferative, and anti-inflammatory properties both in vivo and in vitro [10-15]. Recently, PCA and PAL have been confirmed to have antioxidant effects in many diseases, making these “old compounds” a potential “new application” for medical therapies. However, their antioxidant mechanisms are still not well understood [3]. Here, we aim to fill this gap in knowledge by reviewing the current studies on the anti-oxidative effects and the underlying mechanisms of these compounds in central nervous system-related diseases, cardiovascular diseases, diabetes, liver injury, cancer, obesity, and other diseases and discuss their potential in therapeutic applications.

## 2. Source

### 2.1. Sources of PCA and PAL in Nature

PCA and PAL are widely distributed in nature and are commonly found in vegetables, fruits, plant-derived beverages, and herbal medicines [1, 16]. As shown in Table 1, they are present in rice, crops, and legumes, such as colored rice bran, hemp, and lentils [17-21]. PCA is also found in kidney beans and mung beans [21]. The extract of onion bulbs' external dry layer has been demonstrated to contain quercetin and condensation products of PCA [22]. Basil (*Ocimum basilicum*), lemon thyme (*Thymus citriodorus*), and mint (*Mentha sp*.), belonging to the mint family, which are used as culinary herbs in many countries, contain many antioxidant and anti-inflammatory phenolic compounds such as PCA and PAL among others [23-26]. Fruits and nuts such as friar plum, prune (*Prunus domestica L.*), grapes, gooseberry, currant, and Prunus persica var. platycarpa (*Tabacchiera peach*) also contain PCA and PAL [27-31]. PCA can also be extracted from dried almond hulls (*Prunus amygdalus Batsch*) [32]. Cocoa beans contain 15 phenolic compounds including PCA and PAL [33]. The plant- and fruit-derived products such as barley tea, hot and cold Hibiscus sabdariffa L. (*Hs, roselle; Malvaceae*) beverages [34-37], the crude oil extracted from acai berries (*Euterpe oleracea*) [38], and cultivated Emblica wine [39], and red wine [40] were also found to contain PCA and PAL. The medicinal plants used in traditional Chinese medicines (TCMs) contain the bioactive components PCA and PAL. Ginkgo biloba L [41-43] and Hypericum perforatum [44] contain PCA; Pinellia ternata [45] and *Lilium lancifolium* [46] contain PAL. Some TCMs such as Cynomorium songaricum Rupr., [47] and the fruiting body of Phellinus linteus [48] contain both PCA and PAL. The most famous and one of the most frequently used TCMs is Salvia miltiorrhiza (SM), known as Danshen in Chinese. It contains various phenolic acids and diterpenoids, with relatively higher amounts of PCA 1.43 mg/g and PAL 1.73 mg/g [49]. It has been proved that PAL is an active component of SM and the main degradation product of its water-soluble active component–Salvianolic acid B [50]. The amount of PCA and PAL varies based on the plant part; for example, 0.832 mg/kg fresh weight of PCA is present in Alpinia oxyphylla (AOF) fruit, while about 11.3 mg/kg is found in its air-dried kernels [51, 52].

### 2.2. Sources of PCA and PAL by Metabolism

**Figure 2:**
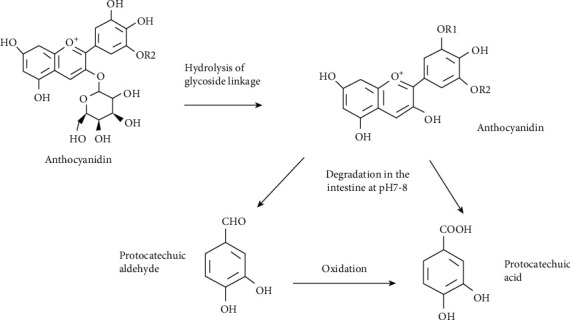
Anthocyanins metabolism into PCA and PAL. A generic anthocyanin with a glucoside moiety is pictured. Parent anthocyanin species are first converted to an aglycon (anthocyanidin) form by hydrolysis of glycoside linkages in the small intestine. PAL could be oxidized to PCA in the intestine.

Gluten-free flours, nuts, fruits, and red wine contain not only dietary antioxidants, such as phenolic acids, flavonoids, and anthocyanins, as has already been described, but they are among the richest food source of bioactive polyphenols (e.g., ellagitannins and proanthocyanidins) [53-55]. Anthocyanins are considered to be the most potent antioxidants among flavonoids [56], and PCA and PAL are the primary metabolites of the complex antioxidant polyphenols, anthocyanins, and proanthocyanidins [53, 54, 57]. The fate of dietary polyphenols was investigated using a simulated in vitro intestinal fermentation system. The food delivers polyphenols to the gastric and intestinal. Digestions do affect the polymeric fractions. The biotransformation of polymerized polyphenols (by gut microbiota) into lower molecular weight compounds, such as caffeic acid, PAL, and PCA, depends on the intestinal phase (pH 6.7–7.4) [53-55]. After absorption, they pass into the bloodstream and are then distributed to the organs, including the brain, to exert their pharmacological and biological effects (Figure 2) [57]. Pharmacokinetic analysis using LC-MS-MS showed that after oral and intravenous administration of PAL into Wistar rats, PAL was extensively metabolized to PCA in the plasma of the rats via oxidation pathways [58, 59]. It was found in the plasma in the form of PAL, PCA, and their conjugates, and the conjugates were detected in the intestine, liver, and kidney. PAL was methylated in the liver, oxidized to PCA, and excreted via urine and bile. A part of the glucuronide conjugates of PAL and PCA excreted into the bile might be converted again to PAL and PCA and reabsorbed in the intestine (Figure 2) [58, 59].

## 3. Mechanism of Antioxidant Effects

**Figure 3:**
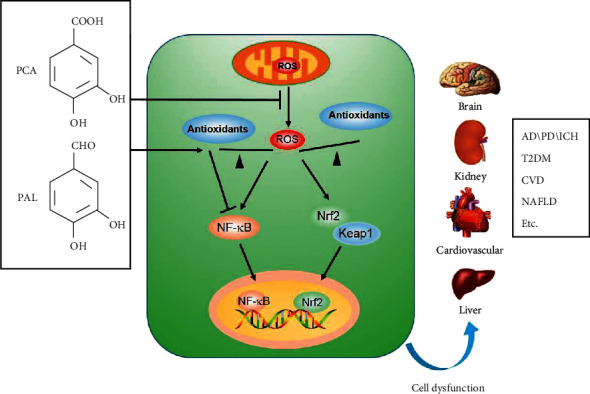
Antioxidant mechanisms of PCA and PAL.

Oxidative stress results from the buildup of reactive oxygen species (ROS) or free radicals, which are the by-products of metabolic processes, and are implicated in the pathogenesis of various diseases including cardiovascular diseases, diabetes, cancer, and neurodegenerative diseases. The antioxidants function through direct or indirect mechanisms including scavenging of ROS and intracellular enzymatic reactions [90]. As they are redox-active with a short life span and are sacrificed when they act on the ROS, they need to be regenerated to curtail the ROS levels. An indirect antioxidant effect can trigger the host cells' self-defense mechanisms to fight oxidative stress. Mitochondria are also morphologically and functionally altered, leading to excessive ROS formation and decrease in energy due to the reduction in ATP production, alteration of calcium homeostasis, and apoptosis induction [91]. The antioxidant potential of PCA and PAL by increasing the activity of endogenous antioxidant enzymes glutathione peroxidase (GSH-PX) and superoxide dismutase (SOD) has been reported in recent studies. PCA is also regarded as the perfect peroxyl radical scavenger in the polar environment of aqueous solutions and a relatively good anti-radical protector in nonpolar environment of lipid solutions [92]. It is capable of attenuating oxidative stress by increasing the activity of GSH-PX and SOD, as well as reducing the activity of xanthine oxidase (XOD) and NADPH oxidase (NOX) and the concentrations of malondialdehyde (MDA) [5, 93]. PAL was reported to inhibit the production of ROS in rat PC12 cells and human SH-SY5Y cells [94, 95]. Tables 2 and 3 provide details of the in vitro and in vivo antioxidant effects of PCA and PAL.  Figure 3 illustrates the antioxidant mechanisms of the 2 metabolites through activation of multiple transcription factors in different cells, tissues, and organs.

### 3.1. Antioxidant Properties in Central Nervous System Diseases

Nerve cells in the brain are metabolically active and vulnerable to injury caused by excessive ROS generation or impairment of the antioxidant defense system, which results in neurodegenerative diseases and brain disorders [96-98]. The antiaging, antioxidant, and antiinflammatory properties of PCA and PAL have been extensively studied in neurodegenerative diseases including Parkinson's disease (PD) and Alzheimer's disease (AD) [3, 4, 99-101], brain injury diseases such as intracerebral hemorrhage (ICH) and cerebral ischemia-reperfusion [104, 105], as well as in depression [106], diabetes-induced oxidative stress in the brain [107], and cadmium (Cd)-induced cortical toxicity [108], in vitro and in vivo.

#### 3.1.1. Neurodegenerative Diseases

Neurodegenerative diseases, including Alzheimer's disease (AD) and Parkinson's disease (PD), have common characteristics consisting of progressive neuronal loss and impaired neuronal function. Oxidative damage, mitochondrial dysfunction, and neuroinflammation are strongly implicated in the pathogenesis of these diseases, which require early intervention to reduce the risks due to the lack of clinically relevant drugs. In the recent times, the use of natural products for the prevention of these diseases has gained importance due to their neuroprotective properties, such as antioxidation and antiinflammation effects [109]. PCA has the potential to counter oxidative stress, excitotoxicity, and neuroinflammatory and nitrosative stress effects [110]. It has been shown to significantly reduce neurodegeneration and locomotor deficits in an MPTP-induced PD mouse model. Several mechanisms may be relevant for bringing about the protective effects. PCA has been reported to increase the nuclear factor erythroid 2-related factor 2 (Nrf2) protein expression and transcription activity; modulate the cellular redox status with the upregulated expression of hallmark antioxidant enzymes, including heme oxygenase-1(HO-1), SOD, and catalase (CAT); and decrease the levels of MDA-treated PC12 cells [102]. Treatment with PCA also inhibited the activation of nuclear factor-κb (NF-κB) and expression of inducible nitric oxide synthase (iNOS) [102]. PCA effectively suppressed palmitic acid (PA)-induced ROS generation and reversed the PA-impaired proliferation of neural stem/progenitor cells (NSPCs) [111, 112]. Furthermore, it demonstrated significant therapeutic effects in preclinical animal models with neurodegenerative diseases such as AD, PD, and D-galactose-induced accelerated aging, in vivo [110, 113, 114]. It could also inhibit the oxidative damage induced by hydrogen peroxide (H_2_O_2_) through the suppression of LDH release, caspase-3 activity, and Bax expression, as well as induction of mitochondrial membrane potential and Bcl-2 expression in cultured retinal ganglion cells (RGC-5) [115]. In cellular models of PD, PAL markedly increased the cell viability rates and mitochondrial oxidation-reduction activity and membrane potential and reduced the intracellular ROS levels in PC12 cells [100]. In animal models of PD, PAL improved specific behavioral defects caused by 6-OHDA and MPTP through its neuroprotective pharmacological effects. The potential mechanisms may be related to the upregulation of the DJ-1 protein, decrease in *α*-synuclein, and its growth-promoting effect on the spine density [100]. PAL could also prevent the tyrosine hydroxylase (TH)-positive dopaminergic neuron loss through induction of DJ-1 and inhibition of *α*-synuclein expression. It also restored oxidative stress-induced inactivation of the Akt pathway and suppressed excessive H_2_O_2_-induced DJ-1 oxidation in SH-SY5Y cells [92]. Post-treatment of PAL in the mouse model of PD showed that it improved the behavioral deficits and protected against dopaminergic neuronal loss [101]. PAL mediated polo-like kinase 2 (PLK2) expression, which upregulated glycogen synthase kinase 3 *β* (GSK3*β*) phosphorylation and Nrf2 nuclear translocation, improved mitochondrial dysfunction, and attenuated oxidative stress injury, which resulted in effective neuroprotection [101]. PCA from Alpinia oxyphylla protected PC12 cells against apoptosis and oxidative stress induced by rotenone by decreasing mitochondrial dysfunction [116]. It reversed the cognitive deficits by inhibiting amyloid deposits and inflammatory response in aged A*β*PP/PS1 double transgenic mice and also recovered the mitochondrial function to counteract the ROS generation in AD [114].

#### 3.1.2. Brain Injury Diseases

PCA had antiapoptotic and antiinflammatory effects and induced antioxidative stress in animal models with intracerebral hemorrhage (ICH) or cerebral ischemia. It improved the endogenous antioxidant defense system by downregulating the P38/JNK-NF-*κ*B pathway in an ICH mouse model and in a rat model of cerebral ischemia [117]. It also attenuated the protein and gene expression of TNF-α, IL-1*β*, and IL-6 in vivo [105]. A single dose of 5 and 10 mg/kg PCA intraperitoneal injection (i.p.) for 7 days in aged rats remarkably led to the induction of CAT and GSH-PX antioxidant activities and suppression of oxidative stress [118]. In experimentally developed chronic intermittent hypoxia rat models, PCA reversed the neurocognitive function impairment through modulation of synaptic plasticity, inhibition of ROS production, and neuronal apoptosis, as well as induction of glial cell proliferation and brain-derived neurotrophic factor (BDNF) expression [93]. Ischemia-induced oxidative stress, activation of astrocytes and microglia, neuronal cell death, blood-brain-barrier (BBB) disruption, and reduction in glutathione (GSH) concentration were reversed by administration of PCA in hippocampal neurons [16]. Seizure-induced hippocampal neuronal death was also inhibited by PCA treatment [119]. PAL too has been reported to protect against cerebral oxidative injury induced by ischemia-reperfusion (I/R) syndrome through PKC*ε*/Nrf2/HO-1 pathway [104].

#### 3.1.3. Other Central Nervous System Diseases

PCA has demonstrated antidepressant potential in stressed animals. It improved endogenous antioxidant enzymatic and antidepressant activities through the upregulation of brain monoamines [118, 120]. PCA (100 and 200 mg/kg body weight) inhibited oxidative activity through suppression of MDA production, induction of enzymatic antioxidant activity, and attenuation of pro-inflammatory cytokines IL-6 and TNF-α in the cerebral cortex and hippocampus in acute restraint stress (ARS) mouse model and olfactory bulbectomized (OBX) mouse model of depression [5, 121, 122]. PCA promoted antioxidant activities, suppressed lipid peroxidation, and decreased the serum corticosterone level in Swiss albino mice with ARS.

PCA alleviated cadmium (Cd)-induced cortical toxicity in male rats through activation of the antioxidant defense system and suppression of the inflammation and apoptosis mediated by the Nrf2/ARE pathway [108]. PCA pre-administration suppressed the cadmium chloride (CdCl_2_)-induced apoptotic events through inhibition of mitochondria-caspase signaling pathway and mitigated Cd-induced neurotoxicity by activating Na+/K+-ATPase, butyrylcholinesterase, acetylcholinesterase, and antioxidant enzymes [124].

Diabetes-induced neurobehavioral and biochemical dysfunction was also alleviated by PCA administration by improving the endogenous antioxidant status; inhibition of lipid peroxidation; and downregulation of acetylcholinesterase, inflammation, and caspase-3 activities [107]. PCA treatment attenuated brain oxidative stress through inhibition of brain mitochondrial dysfunction and hyperglycemia in rats with streptozotocin (STZ)-induced diabetes [4].

### 3.2. Antioxidant Properties in Diabetes

Diabetic cardiomyopathy (DC) is a significant complication in type 2 diabetes mellitus (T2DM). ROS production and oxidative stress in diabetic hearts are being increasingly reported. Excessive oxidative stress correlated with lipid overload, indicating that fatty acid played a role in ROS generation [125]. PCA treatment prevented oxidative damage from lipids and proteins-induced elevation in ROS levels observed in the myocardial tissues of T2D rats. PCA upregulated endogenous antioxidants and recovered the myocardial physiology in these rats through PARP/PKC/NF-*κ*B signaling pathway-mediated anti-inflammatory, antioxidant, insulin-sensitizing, and hypoglycemic effects, which also stimulated glucose metabolism and modulated lipid and glycemic status in the skeletal muscles through IRS1/PI3K/AKT/AMPK/GLUT4/P38 signaling pathways [7]. The diabetes-induced cardiac dysfunction caused by oxidative stress in rats with STZ-induced diabetes was reversed by oral PCA treatment (50 or 100 mg/kg/day) through its antioxidant and antihyperglycemic activities [126, 127]. Several doses of PCA decreased blood glucose and hemoglobin A1c (HbA1c) due to its antihyperglycemic effects [127-129].

In human omental adipocytes, PCA treatment also reversed oxidized low-density lipoprotein (ox-LDL)-induced impairment of glucose uptake through the induction of an insulin-like effect and upregulation of the entire insulin signaling pathway [8, 130, 131]. Oral pretreatment with PCA might counteract insulin resistance status partly through induction of AKT phosphorylation and mitigation of phosphoenolpyruvate carboxykinase (PEPCK) and glucose-6-phosphatase (G6Pase) expression in dexamethasone (Dex)-induced hyperinsulinemia and insulin resistance in rats [132].

PAL was also found to be a potent aldose reductase (AR) inhibitor and might be useful for preventing and/or treating diabetic complications [48, 79]. It showed a significant inhibitory effect on methylglyoxal-mediated protein glycation and protected against protein damage caused by hyperglycemia [133]. As the primary ingredient of Danshen injection (DSI), PAL enhanced the apical-to-basolateral (AP-BL) transport of gliquidone in Caco-2 cell monolayers and increased the absorption of gliquidone [134]. PAL (25 mg/kg body weight) significantly reduced blood glucose levels and inhibited lens opacity in diabetic cataract induced by STZ in rats. The protective mechanism was through inhibition of transforming growth factor (TGF)-*β*1 expression and P-Smad2/3 nuclear accumulation caused by high glucose level or S100B protein in human lens epithelial cells [135].

### 3.3. Antioxidant Properties in Cardiovascular Diseases

PCA has the potential to be a therapeutic candidate for hypertrophic cardiomyopathy as it decreased the 2,3,7,8-tetrachlorodibenzo-p-dioxin (TCDD)-induced cardiotoxicity by suppressing the thiobarbituric acid reactive- substances (TBARS) level and upregulating the GSH-PX, GSH, SOD, and CAT levels in the rat heart tissue [136]. It suppressed cleaved caspase-3 expression and reduced hypoxia/reoxygenation-induced cardiomyocyte apoptosis rate [136]. Treatment with PCA and glibenclamide counteracted oxidative damage by suppressing total cholesterol (TC), low-density lipoprotein cholesterol (LDL-C), very low-density lipoprotein cholesterol (VLDL-C), triglyceride (TG), and lipid peroxidation markers and reduced diabetes-induced cardiac dysfunction [127, 138]. PCA administra-tion for 12 weeks augmented insulin and IGF-1-induced endothelium-dependent vasorelaxation in spontaneously hypertensive male rats by activating the PI3K-NOS-NO pathway [139]. PCA showed hypoglycemic, insulin-sensitizing, hypolipidemic, and antioxidant effects in glucocorticoid (GC)-induced hypertensive rats [140]. It prevented hypertension and weight loss, reduced plasma H_2_O_2_ concentration, and increased ferric reducing antioxidant power (FRAP) values in a dose-dependent manner [140]. Furthermore, PCA showed antihypertensive and antioxidant effects against Dex-induced hypertension by improving the antioxidant capacity and reducing the blood pressure [132]. It also mitigated vascular dysfunction induced by Dex and enhanced the relaxation in aortic rings induced by acetylcholine (ACh), through induction of endothelial nitric oxide syn-thase (eNOS) and reduction of NADPH oxidase (NOX4) expression [132]. PAL was also shown to provide relief from atherosclerosis by inhibiting lipopolysaccharide (LPS)-induced endothelial apoptosis. It inhibited the JAK2/STAT3 signaling pathway and protected against cardiac hypertrophy induced by isoproterenol (ISO) and effectively suppressed myocardial fibrosis in ISO-induced heart failure mouse model [141]. It provided cardioprotection against myocardial ischemia/reperfusion injury in rats by suppressing NF-κB-mediated inflammatory response [11]. Moreover, researchers observed that PAL bound to collagen I protein through lysine residues via covalent crosslinking, making it a crucial pharmacological target for treating myocardial fibrosis [142]. It showed inhibitory effects against angiogenesis by increasing CD31 and GPER-1 and decreasing VCAM-1 and CD40 expression aortic ring assay in rats [143]. PAL protected SH-SY5Y cells from H_2_O_2_-induced oxidative stress through stimulation of antioxidants (SOD2 and CAT) and B-cell lymphoma 2 (Bcl-2), as well as forkhead box O (FoxO) 3a and sirtuin-1 (SIRT1) [144].

A study noted that PCA could induce HO-1 expression and increase SOD and GSH-PX 1 (GPx-1) activities through the LKB1-AMPK-Nrf2 pathway in human umbilical vein endothelial cells (HUVECs) in vitro [145]. The same group also found that PCA deacetylation played a role in manganese-dependent SOD (MnSOD), as well as Ac-CoA generation or SIRT1 and SIRT3 activation through the CD36/AMPK signaling pathway [146]. Research has shown that due to its location in the mitochondria, MnSOD plays a significant role as a ROS scavenger, whereas SIRT1 and SIRT3 are vital for the control of metabolic processes. PCA prevented apoptosis induced by oxLDL by activating JNK/Nrf2 survival signals in J774A.1 macrophages, an in vitro model for investigating pathological atherosclerosis processes [147]. PAL is capable of inhibiting vascular smooth muscle cells' (VSMCs) proliferation and migration due to its ROS scavenging ability through attenuation of platelet-derived growth factor (PDGF) and other cytokines' signaling cascades [15]. It protects endothelial cells from inflammation by attenuating G protein-coupled estrogen receptor-1 (GPER-1)-mediated endothelial dysfunction [143, 148].

### 3.4. Antioxidant Properties in Liver Injury

PCA prevented ROS formation in alcoholic liver disease (ALD) by stimulating the expression of miR-219a-5p (a tumor suppressor) and repressing p66Shc (an adaptor protein involved in ROS formation in mitochondria) [149]. PCA treatment in nonalcoholic fatty liver disease (NAFLD) activated SIRT3 partly by suppressing fatty acid metabolism, mediated by ACSF3 [150]. It was effective in the treatment of I/R-induced liver injury through inhibition of p66Shc in mice and also in restoring D-galactosamine- or cadmium-induced fatty liver in rats [151-153]. PCA inhibited Dex-induced liver steatosis by reducing serum aspartate aminotransferase (AST) and alanine aminotransferase (ALT) activity. PCA counteracted hepatotoxicity induced by t-BHP by blocking MDA and ROS generation or stress-induced changes in enzyme activity [88].

PCA demonstrated antioxidative and antiapoptotic effects on I/R-induced liver injury in Caco-2 cell line by suppressing p66Shc [151]. It also inhibited hepatitis B virus replication in HepG2 2.2.15 cell line [154] and also hampered the catalytic activity of cytochrome P450 enzyme by downregulating CY3A4 in human liver microsomes [155]. PCA and PAL counteracted H_2_O_2_-induced hemolysis of rat erythrocytes through inhibition of MDA production in liver microsomes and peroxidative damage to the hepatocytes' surfaces [156].

### 3.5. Antioxidant Properties in Cancer

As a significant anthocyanin metabolite in dietary polyphenol-rich food, the antioxidative effects of PCA were evident during cancer progression in humans [157]. It could induce cell death through suppression of Bcl-2 expression and retinoblastoma (RB) phosphorylation in human leukemia cells and c-Jun N-terminal kinase-dependent signal transduction in HepG2 hepatocellular carcinoma cells [158, 159]. The anthocyanin and PCA present in black raspberries suppressed esophageal tumorigenesis induced by N-nitroso-methyl benzylamine (NMBA), through inhibition of COX-2, iNOS, p-NF-kB, and sEH biomarkers and PTX3 cytokine expression [160]. Similarly, PAL also showed anti-tumor activity by hindering the proliferation of MCF-7 human breast cancer cells through activation of DPPH radical-scavenging activity [161].

An effective cancer therapy strategy involves manipulation of the ROS levels. Pre-treatment with PCA inhibited DBP-diol-induced DNA adduct formation and mutagenesis through enzyme inhibition or phase 2 enzyme induction [162]. PCA inhibits ovarian cancer cells proliferation through p53-independent apoptosis and autophagy induction along with ROS reduction [163]. PAL suppressed cisplatin-induced acute kidney injury (AKI) by inhibiting NOX-mediated renal inflammation and oxidative stress, making PAL and its derivatives potential chemoprotective agents [164]. It also suppressed breast cancer cell growth and promoted apoptosis of these cells by downregulating b-catenin- and cyclin D1-mediated multiple signal pathways [34].

### 3.6. Antioxidant Properties in Other Diseases

PCA counteracted TCDD-induced reproductive damage in male rats. The use of PCA in combination with TCDD minimized the latter's toxicity through suppression of histopathological changes and TBARS levels and increasing SOD, CAT, GPx antioxidant enzymes activity; GSH levels; and sperm concentration and motility [165]. PCA also showed a protective effect against nephrotoxicity resulting from doxorubicin (DOX)-induced damage to the renal cells [166]. Pretreatment with PCA prior to the administration of DOX reduced the MDA levels, modulated iNOS and cyclooxygenase-2 (COX2) activities, and improved renal function, as well as enhanced the antioxidant parameters including GSH level and activities of the antioxidant enzymes SOD, CAT, GSH-PX and glutathione s-transferase (GST) in the kidney [166].

PCA protected renal I/R injury by suppressing oxidative stress and tissue damage [167]. It significantly controlled the serum levels of MDA and TNF-α, and renal MDA level; while on the other hand, it upregulated the serum and renal total antioxidant status (TAS) and SOD levels, and histopathological scores [167]. PCA administration for 28 days suppressed benign prostatic hyperplasia induced by testosterone through reduction of myeloperoxidase (MPO) activity, and NO and MDA levels. Furthermore, it restored the histological architecture of the prostate epithelium in rats with benign prostatic hyperplasia (BPH) [168].

The acute lung injury (ALI) induced by lipopolysaccharide (LPS) in mice was repaired by PCA by downregulating p38/MAPK and NF-κB signal pathways [169]. It controlled dermal wounds in Wistar albino rats by elevating the levels of SOD, CAT, and GPx antioxidant enzymes and suppressing the inflammatory markers [170]. PAL inhibited TGF-β1–stimulated epithelial-mesenchymal transition (EMT) in A549 cells and controlled bleomycin (BLM)-induced pulmonary fibrosis in rats through HMGB1/RAGE pathway regulation [9].

Pretreatment with PCA-enhanced ROS generation, lipid peroxidation, and DNA fragmentation; upregulated SOD, CAT, NAD+/NADH levels; and downregulated GSH and MDA levels—consequently leading to bacterial cell death in E. coli, P. aeruginosa, and S. aureus probably through Fenton chemistry, autoxidation, and electron transport chain inhibition [171]. Dose-dependent PCA administration reduced multinucleated osteoclasts formation and tartrate-resistant acid phosphatase (TRAP) activity in RANKL-treated RAW264.7 murine macrophage cells through a decrease in the ROS and lipid peroxide levels and enhancement of the antioxidant status [172]. PCA suppressed the expression of osteoclast-specific markers such as Cathepsin, MMP, c-Src, TRAP, TRAF-6, and transcription factors AP-1 and NFATc1; MAPK activation; as well as NF-kB and COX-2 inflammatory marker levels [172]. PAL could also reduce LPS-induced NO production, stimulate free radical scavenging activity in RAW264.7 macrophages [12], block NF-κB activation, and decrease HMGB1 expression during cecal ligation and puncture (CLP)-induced sepsis [14]. In osteoarthritis (OA) induced by anterior cruciate ligament transection (ACLT), PCA suppressed osteoclastogenesis by modulating the MAPK, ATK, and NF-κB signaling pathways [173]. It also altered the aging rate and life span of the nematode, Caenorhabditis elegans. It alleviated intracellular ROS level and CAT and SOD antioxidant enzyme activities [120].

## 4. Clinical Studies of PCA and PAL in TCM

PCA and PAL are the active ingredients in many traditional Chinese medicines, such as Salviae miltiorrhiza (Danshen) and Acanthopanax senticosus (Ciwujia). They were also detected in some proprietary traditional Chinese herbal injections, such as Danshen injection (DSI), Compound Danshen injection (CDSI), Xiangdan injection (XDI), Guanxinning injection (GXN), Danhong injection (DHI), and XueBiJing injection (XBJ). These have been widely used in the treatment of cardiovascular (such as acute coronary syndrome and angina pectoris) and cerebrovascular diseases (such as stroke) in China for many years. Their pharmacological properties related to PCA and PAL include antioxidant, anticoagulatory, hypolipidemic, antiapoptotic, antiinflammatory, vasodilatory, and angiogenesis-promoting actions [175].

DSI is made from only Salviae miltiorrhiza, whereas CDSI and XDI are made up of Danshen and Dalbergia odorifera (Jiangxiang). DHI consists of two famous Chinese herbal medicines, Danshen and Carthami flos (Honghua) [176]; GXN is prepared from Danshen and Rhizoma Chuanxiong (Chuan xiong). According to related studies, the main effective constituent of these injections is Danshen, which contains high levels of PCA and PAL [175, 177-180]. Similarly, in XBJ, a total of 17 Danshen catechols were detected, among which the amount of PCA and PAL was high [181]. Acanthopanax senticosus extract injection (ASI) also contains many ingredients including PCA [182]. PAL can be largely oxidized to PCA in vivo, which is an abundant circulating metabolite despite its low content in the injection. Although some injections are composed of several medicines besides Danshen, they do not affect the main components of Danshen. A comparative pharmacokinetic study suggested that the presence of Honghua constituents along with Danshen in DHI might have negligible influences on the pharmacokinetics of the polyphenols derived from Danshen [175].

### 4.1. Antioxidant Effect on Central Nervous System Disease

DHI protects patients from cerebral injury during coronary artery bypass graft (CABG) surgery with cardiopulmonary bypass (CPB) through antioxidation, antiinflammation, and immune factors regulation mechanisms [183, 183]. Composite salvia injection (CSI) has the potential to reduce the oxygen-free radical damage and regulate the apolipoprotein metabolism in patients with ischemic cerebral infarction by decreasing the serum levels of lipid peroxide (LPO) and apolipoprotein B100 (APOB100) and increasing the SOD and ApoA1 levels [185]. It has also been noted to effectively reduce the serum levels of MPO and hypersensitive C-reactive protein (hs-CRP) in patients with severe preeclampsia (PE) [186].

### 4.2. Antioxidant Effect on Type 2 Diabetes Mellitus (T2DM) Diseases

CWJI has a protective effect in type 2 diabetes (T2DM) patients with persistent microalbuminuria and normal blood pressure by inhibiting kidney endothelin (ET) synthesis, reducing the level of urinary albumin excretion (UAE), plasma ET concentration, and urinary ET excretion [187]. CSI in combination with western medicine (WM) was found to be effective in the treatment of diabetic foot by accelerating the median motor nerve and sensory nerve conduction speed and lowering the blood viscosity [188].

### 4.3. Antioxidant Effect on Cardiovascular Diseases

GXN is widely used to treat angina, hyperlipidemia, and coronary heart disease [189, 190]. When patients with chronic stable angina were treated with DHI, they showed a clinically significant change, defined as at least a 20-point improvement in the angina frequency score on the Seattle Angina Questionnaire. The other secondary efficacy and safety outcomes were also assessed at the same time [191]. DSI decreased the endothelin-1 (ET-1) response and increased postoperative hemodynamic stability; reduced myocardial damage; and corrected the imbalance in the levels of vasoactive mediators after surgery in children with congenital heart defects [192]. It improved the plasma (ET-1), nitric oxide (NO), and apolipoprotein levels and effectively relieved the clinical symptoms of patients with arteriosclerosis obliterans (ASO) [120]. Shenmai-Danshen (SM-DS) injection im-proved MDA, SOD, interleukin 6 (IL-6), and TNF-alpha levels and reduced the myocardial reperfusion injury in patients with acute myocardial infarction, after percutaneous coronary intervention (PCI) [193]. The level of plasma ET-1, sP-sel, and hs-CRP following treatment with DHI indicated that it could inhibit platelet activation and inflammation, and angiogenesis and protect the endothelial function in patients with acute coronary syndrome (ACS) after PCI [194-198].

### 4.4. Antioxidant Effect on Liver Diseases

DSI was effective in the treatment of liver cirrhosis and fibrosis due to chronic hepatitis B virus infection [199-201], and intrahepatic cholestasis (ICP) by inhibiting ROS production [202].

XBJ prevented the release of serum proinflammatory cytokines, thereby alleviating hepatic I/R injury and promoting the recovery of intestinal function, but it did not demonstrate a protective effect on coagulopathy [203].

### 4.5. Antioxidant Effect on Cancer

Treatment with ASI significantly improved the activity of human tumor necrosis factor-beta (TNF-beta) and the levels of IgA, IgG, and IgM, as well as the activity of natural killer (NK) cells in patients with lung cancer [204].

### 4.6. Antioxidant Effect on Other Diseases

When critically ill patients with severe community-acquired pneumonia were given XBJ, there was a significant improvement in the pneumonia severity index, primary end points, secondary clinical outcomes of mortality, and duration of mechanical ventilation and ICU stay [205].

When COVID-19 patients were treated with XBJ, the inflammatory markers such as white blood cell count, lymphocyte count, C-reactive protein level, and erythrocyte sedimentation rate significantly increased. The condition and prognosis of the patients improved [206]. XBJ is also effective in the treatment of patients with severe acute pancreatitis (SAP). It could decrease the incidence of complications, shorten the length of stay in hospitals, and reduce the mortality rate in SAP patients [207].

## Figures and Tables

**Table 1:** Sources of PCA and PAL in nature and their biological activities.

No.	Biological source	PCA content (ug/g)	PAL content (ug/g)	Biological activity	References
1	Rice	23.2–1043 (DW)	2–188 (DW)	Antioxidative, anti-inflammatory chemoprevention	[18, 60, 61]
2	Buckwheat (*Fagopyrum esculentum*)	6.61–24.5 (DW)	3.65–19.74 (DW)	Antioxidative	[21, 62]
3	Green pea (*Pisum sativum*)	1.26–11.38 (DW)	0.07–0.12 (DW)	Antioxidative	[21, 63]
4	Fava bean (*Vicia faba*)	0.61–2.42 (DW)	0.68–5.63 (DW)	NT	[21]
5	Hemp (*Cannabis sativa*)	5.63–22.06 (DW)	6.41–34.77 (DW)	NT	[21]
6	Lupin (*Lupinus albus*)	0.15 ± 0.02 (DW)	ND	NT	[21]
7	Wheat	0.07–0.11 (DW)	0.19 ± 0.04 (DW)	Antioxidative	[21]
8	Lentils	20.28–37.72 (DW)	3.69–12.14 (DW)	Antioxidative, anti-inflammatory	[20]
9	Commercial black-colored cowpeas	18.97 ± 0.45 (DW)	NT	Antioxidative antidiabetic	[64]
10	Pea (*Pisum sativum* L.) varieties	12.1–163.5 (DW)	NT	Antioxidative, anti-inflammatory immunomodulation	[65]
11	Common beans	95.34–253.42 (DW)	NT	Antioxidative	[66]
12	Onion (*Allium cepa* L.)	1027 (DW)	NT	Antioxidative, antimutagenic	[22]
13	Mint family plants	UC (FW)	0.843–18.285 (FW)	Antioxidative, anti-inflammatory	[26]
14	Yayla Cayi (*Thymus praecox* OPIZ subsp. Grossheimii (Ronniger) Jalas)	UC (DW)	UC (DW)	Antioxidative	[67]
15	Loquat (*Eriobotrya japonica* L.)	0.843–18.285 (DW)	NT	Antioxidative	[68]
16	Kinnow peel	177 (DW)	NT	Antioxidative, health benefits	[69]
17	Banana pulp	340 (DW)	NT	Antioxidative, health benefits	[69]
18	Prune (*Prunus domestica* L.)	355.87 (FW)	NT	Antioxidative	[27]
19	Friar plum (*Prunus salicina* Lindl.)	50–160 (FW)	NT	Antioxidative, increased edible quality	[28]
20	*Prunus persica* var. platycarpa (Tabacchiera peach)	0.19 (FW)	0.02 (FW)	Antioxidative	[31]
21	Currant (*Ribes* L.)	137.6–464.8 (FW)	NT	Antioxidative	[30]
22	Gooseberry (*Ribes uva-crispa* L.)	24.7–77.7 (DW)	NT	Antioxidative	[30]
23	Grapes	0.143–0.371 (FW)	NT	Antioxidative	[29]
24	Acai (*Euterpe oleracea* Mart.) seed	106–843 (DW)	NT	Antioxidative, antimalarial, antiplasmodial	[70]
25	Cocoa beans	197.9–385.3 (DW)	2.6–1945.7 (DW)	Antioxidative, anti-inflammatory	[33]
26	Almonds (*Prunus amygdalus* Batsch)	66.67 (DW)	NT	Antioxidative	[71]
27	Pecan (*Carya illinoinensis*)	13.1–30.5 (FW)	UC	Antioxidative	[72]
28	*Orobanche cernua* Loefling (Orobanchaceae)	NT	0.353 (FW)	Anticancer	[73]
29	*Salvia miltiorrhiza*	56～152 (DW)	59～94 (DW)	Antioxidative, anti-inflammatory	[49]
30	The fruiting body of *Phellinus linteus*	10 (FW)	9 (FW)	Aldose reductase inhibitors	[48]
31	*Ginkgo biloba* L. leaf	2708～345321 (FW)	NT	Antioxidative	[42, 43]
32	*Aesculus hippocastanum* L. (Hippocastanaceae)	72.53 (DW)	NT	Antioxidative	[44]
33	*Hypericum perforatum*	761.67 (DW)	NT	Antioxidative, anti-inflammatory, antigenotoxic	[44]
34	Alpinate oxyphyllae fructus (*Alpinia oxyphylla* MIQ, AOF)	0.832 (FW), 8.5 (DW)	NT	Antioxidative, anti-cell migration, antiapoptosis	[51, 74]
35	*Schisandra chinensis* (Turcz.) Baill. fructus (SCF)	210 (DW)	NT	Antioxidative, anti-beta amyloid formation	[75, 76]
36	Ramulus Cinnamomi	61.5～137.7 (DW)	NT	Antioxidative	[77]
37	Cinnamon fruits	54.7 (DW)	NT	Antioxidative	[78]
38	*Lilium lancifolium*	0.5937～2.962 (DW)	NT	Antioxidative	[46]
39	*Cynomorium songaricum* Rupr.	148 (DW)	0.629 (DW)	Phytoestrogenic- or phytoandrogenic-like activities	[47]
40	The fruiting bodies of *Ganoderma lucidum*	NT	0.952 (DW)?	Aldose reductase inhibitors	[79]
41	*Pinellia ternata*	NT	UC	Antioxidative, change of activity of protective enzyme	[45, 80]
42	*Prunella vulgaris*	0.0089～0.0476% (w/w)	0.003～0.008% (w/w)	Antioxidative	[81]
43	Black cohosh (*Actaea racemosa* L.)	8.8 (DW)	4.6 (DW)	Antioxidative, anticancer	[82]
44	Rattan materials (*Calamoideae faberii*)	14～97 (DW)	18～99 (DW)	Antioxidative	[83]
45	Leaves of *Lycium barbarum*	NT	0.87～9.47 (DW)	Antioxidative	[84, 85]
46	*Hydnophytum formicarum* Jack. (Rubiaceae)	NT	1.5 (DW)	Antioxidative, antimicrobial	[86]
47	*Hibiscus sabdariffa* L. (*Hs*, Roselle; Malvaceae)	94.1 (DW)	NT	Antioxidative, anti-inflammatory, antiurease	[87, 88]
48	Echinacea (*Echinacea* purpurea)	UC	UC	Antioxidative, anti-inflammatory	[67]
49	Green tea (*Camellia sinensis*)	UC	UC	Antioxidative, anti-inflammatory	[67]
50	Barley tea	NT	2.6 (DW)	Antioxidative, anti-inflammatory	[89]
51	Grape wine	0.7～5.24 mg/L	NT	Antioxidative, anti-inflammatory	[39, 40]
52	Olive oil (*Olea europaea*)	176.08 (DW)	NT	Antioxidative, anti-inflammatory	[44]

DW, dry weight; FW, fresh weight; ND, not detected (i.e., below the detection level); NT, not tested; UC, detected, but content unclear.

**Table 2:** Summary of the effects on antioxidants of PCA and PAL in vitro.

Disease model	PCA/PAL dosages	Model used	Oxidative stress mechanisms	References
Neurodegenerative diseases	PCA (50, 100, 150, and 200 *μ*g/ml)	H_2_O_2_-treated PC12 cells	Downregulation of lipid peroxidation and upregulation of glutathione peroxidase and superoxide dismutase activity	[118]
PD	PAL (20 *μ*M)	H_2_O_2_-treated SH-SY5Y cells	Activation of the Akt pathway and suppression of excessive DJ-1 oxidation	[95]
PD	PCA (1 mM)	6-OHDA-treated PC12 cells	Activation of Nrf2/HO-1 and suppression of NF-*κ*B signaling	[102]
PD	PCA (10 *μ*M)	MPP-treated SH-SY5Y cells	Mitigation of oxidative damage and mitochondrial dysfunction through PLK2/p-GSK3*β*/Nrf2 pathway	[101]
PD	PAL (1, 10, and 100 *μ*M/0.1, 1, and 10 *μ*M)	H_2_O_2_/6-OHDA-treated PC12 cells	Induction of DJ-1 and reduction of *a*-synuclein expression	[100]
Diabetic cataract	PAL (20 and 50 *μ*g/ml)	High glucose- or S100b-treated human lens epithelial cells	Inhibition of TGF-*β*1 expression and pSmad2/3 nuclear accumulation	[135]
Cerebral I/R injury	PAL (80 *μ*M)	Differentiated SH-SY5Y cells	Induction of Nrf2 nuclear translocation and HO-1 upregulation	[104]
CVD	PCA (10, 50, and 100 *μ*M)	Palmitic acid- (PA-) treated HUVECs.	Suppression of Ac-CoA or Sirt1 and Sirt3 activation through CD36/AMPK signaling	[146]
CVD	PAL (100 *μ*M)	Thoracic aortic smooth muscle cells	Inhibition of PDGF and other cytokines cascade signaling	[15]
CVD	PCA (10–100 *μ*M)	PA-treated HUVECs	Induction of HO-1 and increasing SOD and GPx-1 activity through LKB1-AMPK-Nrf2 pathway	[145]
Atherosclerosis	PAL (10, 50, or 100 *μ*M)	HUVECS	Reduction of ROS activity and inflammation, increase of cAMP and GPER-1	[143]
Ischemic injury	PCA (10, 20, and 40 *μ*M)	H_2_O_2_ treat H9C2 cells	Reduction of ROS and elevation of GSH	[148]
Liver injury	PCA (2.5, 5, and 10 *μ*M)	PA-treated AML-12 cells	Activation of SIRT3 and suppression of ACSF3-mediated fatty acid metabolism disorder	[150]
Liver injury	PCA (10 *μ*M)	Primary hepatocytes, AML-12 cells	Upregulation of miR-219a-5p expression and suppression of p66shc-mediated ROS formation	[149]
Liver injury	PCA 20 ug/ml, PAL 20 ug/ml	Ccl4 treatment isolated rat hepatocytes	Inhibit MDA production in liver microsomes and peroxidative damage to the surfaces of rat hepatocytes.	[156]
Cancer	PCA (500 ppm)	N-nitroso-methyl benzylamine- (NMBA-) induced esophageal tumorigenesis	Inhibition of tumorigenesis and inflammatory signaling	[160]
Cancer	PCA (100 *μ*M)	HepG2 hepatocellular carcinoma cells	Induction of JNK-dependent hepatocellular carcinoma cell death	[159]
Cancer	PAL (0.25, 0.5, and 1 *μ*M)	Cisplatin-treated HK2 cells	Suppression of cisplatin-induced death through inhibition of Nox4	[164]
Pulmonary injury	PCA (25 *μ*M)	Fluoride-treated A549 cells	Inhibition of fluoride toxicity through regulation of intracellular calcium level, bioavailability, redox signaling, and mitochondrial membrane integrity	[174]

**Table 3:** Summary of the effects on antioxidants of PCA and PAL in vivo.

Diseases	PCA/PAL dosages	Model used	Oxidative stress mechanisms	References
Neurodegenerative diseases	PCA (5 mg/kg/day, i.p.)	Male SD rats	Downregulation of lipid peroxidation and upregulation of glutathione peroxidase and superoxide dismutase activity	[118]
PD	PAL mice (7.5, 15, and 30 mg/kg/12 h p.o.) and rats (20 and 40 mg/kg/12 h, p.o.)	MPTP-induced PD in mice and 6-OHDA-induced SD rats	Induction of DJ-1 and reduction of *a*-synuclein expression	[100]
PD	PCA (10 and 20 mg/kg/day, i.p.)	MPTP-intoxicated mice	Mitigation of oxidative damage and mitochondrial dysfunction through PLK2/p-GSK3*β*/Nrf2 pathway	[101]
PD	PCA (6, 12, and 25 *μ*M)	6-OHDA-induced PD zebrafish	Downregulation of lipid peroxidation and induction of antioxidant enzymes (SOD, CAT, and GSH) activity	[102]
Cerebral ischemic	PCA (200 mg/kg/day, i.p.)	Cerebral ischemic rats	Suppression of MDA formation and induction of endogenous antioxidant (SOD, CAT, and GSH) activity	[117]
Depression	PCA (100 and 200 mg/kg/day, p.o.)	OBX induced depressive-like behavior in Wistar rat model	Induction of BDNF, 5-HT, DA, and NE and suppression of MDA, IL-6, and TNF-*α*	[121]
Depression	PCA (100 and 200 mg/kg/day, p.o.)	ARS-induced depressive-like behavior in Swiss albino mice	Induction of antioxidant marker levels/activities	[106]
I/R injury	PAL (40 mg/kg/day, i.v.)	Middle cerebral artery occlusion- (MCAO-) induced I/R injury in SD rats	Activation of PKC*ε*/Nrf2/HO-1 pathway	[104]
Diabetes	PCA (50, 100, and 200 mg/kg/day, p.o.)	STZ-diabetic rats	Induction of antihyperglycemic effect	[128]
Diabetes	PCA (50 and 100 mg/kg/day, p.o.)	T1DM adult male SD rats	Inhibition of brain oxidative stress by improving brain mitochondrial function in T1DM rats	[91]
Diabetes	PCA (50 and 100 mg/kg/day, p.o.)	DC-treated Wistar rats	Induction of antioxidant, hypoglycemic, insulin-sensitizing, and anti-inflammatory effects	[7]
Diabetes	PCA (50 mg/kg/day, p.o.)	STZ-diabetic rats	Induction of endogenous antioxidant, suppression of lipid peroxidation, inflammation, caspase-3, and acetylcholinesterase activities	[107]
CVD	PCA (50, 100, and 200 mg/kg/day, p.o.)	Glucocorticoid-induced hypertension in rats	Suppression of plasma H_2_O_2_ concentration and induction of FRAP values	[140]
CVD	PCA (200 mg/kg/day, p.o.)	Male aging spontaneously hypertensive rats	Induction of insulin and IGF-1 in aging hypertension by PI3K-NOS-NO signaling	[139]
CVD	PCA (100 mg/kg/day, p.o.)	TCDD-induced cardiotoxicity in male SD rats	Suppression of oxidative stress	[136]
CVD	PCA (50 and 100 mg/kg/day, p.o.)	Dex-induced hypertensive male Wistar rats	Induction of eNOS and suppression of NOX4 expression	[132]
CVD	PCA (100 mg/kg/day, p.o.)	HFD-fed male C57BL/6J mice	Suppression of Ac-CoA or Sirt1 and Sirt3 activation through CD36/AMPK signaling	[146]
CVD	PAL (10, 30, and 100 mg/kg/day, i.g.)	Isoproterenol-induced cardiac hypertrophy in SD rats	Inhibition of JAK2/STAT3 signaling	[141]
CVD	PAL (100 mg/kg/day, p.o.)	Common Carotid Balloon injury SD rats	Reduction of ROS activity and inflammation, increase of cAMP and GPER-1, increased CD31 and GPER-1, and decreased VCAM-1 and CD40 expression	[143]
Myocardial fibrosis	PAL (30 mg/kg/day, i.p.), PCA (30 mg/kg/day, i.p.)	Isoprenaline- (ISO-) treated C57BL/6 mice	Modulation of collagen conformational dynamics	[142]
Ischemic injury	PCA (4 mg/kg, i.p.)	I/R injury SD rats	Elevation of GSH levels and reduction of ROS	[148]
Obesity	PCA (100 *μ*M)	Visceral adipose tissue from female obese individuals	Suppression of PTP1B activity	[8]
NAFLD	PCA, rats (10 and 20 mg/kg/day), mice (30 mg/kg/day)	HFD-fed male SD rats, C57BL/6 mice, and SIRT3−/− mice	Suppression of NAFLD through the SIRT3/ACSF3 signaling	[150]
Liver injury	PCA (0.45, 0.9, and 1.8 mg/kg/day, i.p.)	Cisplatin-induced acute kidney injury in mice	Suppression of cisplatin-induced death through inhibition of Nox4	[164]
Liver injury	PCA (50 and 100 mg/kg/day, p.o.)	t-BHP-treated male SD rats	Induction of antioxidant enzymatic activities and suppression of stress signal transduction	[88]
Liver injury	PCA (10 and 20 mg/kg/day, p.o.)	ALD male SD rat model	Induction of miR-219a-5p expression and suppression of p66shc-mediated ROS formation	[149]
Liver injury	PCA (100 mg/kg/day, p.o.)	D-GalN-induced hepatotoxicity in male albino Wistar rats	Induction of antihyperlipidemic activity and DNA damage protection	[152]
Liver and kidney injury	PCA (10 and 20 mg/kg/day, p.o.)	Cadmium-induced hepatotoxicity and nephrotoxicity in male Wistar rats	Suppression of lipid peroxidation and elevation of kidney parameters and liver marker enzymes	[153]
Kidney injury	PCA (10 and 20 mg/kg/day, i.p.)	DOX-treated Wistar rats	Reversion of kidney antioxidant enzymes CAT, SOD, GPx, GSH, and GST levels	[166]
Aging	PCA (100 and 200 *μ*M)	Age-synchronized N2 *Caenorhabditis elegans*	Inhibition of intracellular ROS level and antioxidant enzyme activities of nematodes	[120]
Reproductive damage	PCA (10 and 40 mg/kg/day, p.o.)	Testosterone propionate- (TP-) induced BPH castrated albino Wistar rat	Reduction of MPO activity, MDA, and NO level	[168]
Sepsis	PAL (25, 50, and 100 mg/kg, injection into the tail vein)	CLP-induced sepsis in male Sprague-Dawley rats	Suppression of HMGB1 and NF-*κ*B signaling	[14]

## Data Availability

No data were used to support this study.
